# NADPH oxidase-generated reactive oxygen species in mature follicles are essential for *Drosophila* ovulation

**DOI:** 10.1073/pnas.1800115115

**Published:** 2018-07-09

**Authors:** Wei Li, Jessica F. Young, Jianjun Sun

**Affiliations:** ^a^Department of Physiology & Neurobiology, University of Connecticut, Storrs, CT 06269;; ^b^Institute for Systems Genomics, University of Connecticut, Storrs, CT 06269

**Keywords:** NADPH oxidase, superoxide dismutase, hydrogen peroxide, ovulation, octopamine

## Abstract

Ovarian reactive oxygen species (ROS) are believed to regulate ovulation in mammals, but the details of ROS production in follicles and the role of ROS in ovulation in other species remain underexplored. In *Drosophila* ovulation, matrix metalloproteinase 2 (MMP2) is required for follicle rupture by degradation of posterior follicle cells surrounding a mature oocyte. We recently demonstrated that MMP2 activation and follicle rupture are regulated by the neuronal hormone octopamine (OA) and the octopamine receptor in mushroom body (OAMB). In the current study, we investigated the role of the superoxide-generating enzyme NADPH oxidase (NOX) in *Drosophila* ovulation. We report that *Nox* is highly enriched in mature follicle cells and that *Nox* knockdown in these cells leads to a reduction in superoxide and to defective ovulation. Similar to MMP2 activation, NOX enzymatic activity is also controlled by the OA/OAMB-Ca^2+^ signaling pathway. In addition, we report that extracellular superoxide dismutase 3 (SOD3) is required to convert superoxide to hydrogen peroxide, which acts as the key signaling molecule for follicle rupture, independent of MMP2 activation. Given that *Nox* homologs are expressed in mammalian follicles, the NOX-dependent hydrogen peroxide signaling pathway that we describe could play a conserved role in regulating ovulation in other species.

## Significance

Reactive oxygen species (ROS) cause oxidative stress and damage in many pathological conditions, but they can also function as signaling molecules in physiological processes. It is difficult, however, to decipher where ROS come from and which ROS are involved in these processes. In this article, we demonstrate that a NADPH oxidase (NOX) and an extracellular superoxide dismutase (SOD3) function in follicle cells of *Drosophila* egg chambers to produce hydrogen peroxide, which regulates follicle rupture and ovulation, a process essential for reproduction. NOX and SOD3 are expressed in human follicles and could potentially play similar roles in humans. Our work thus provides potential targets for treating ROS-related infertility or developing novel contraceptive approaches.

Ovulation is a key step in animal reproduction and involves multiple endocrine, paracrine, and autocrine signaling molecules, such as progesterone, epidermal growth factors, and prostaglandins. These molecules ultimately activate proteinases that break down the ovarian follicle wall, releasing a fertilizable oocyte (1–3). Several lines of evidence indicate that reactive oxygen species (ROS) also play indispensable roles in mammalian ovulation (4–8). However, there is no genetic evidence to support an in vivo role of ROS in ovulation, and the enzymes responsible for ROS production during ovulation are still unknown.

ROS are oxygen-derived, chemically reactive small molecules and include superoxide anion (O_2_^•−^), hydrogen peroxide (H_2_O_2_), and hydroxyl radicals (OH•) (9). The physiological generation of ROS can occur as a byproduct of aerobic metabolism or as the primary function of the family of NADPH oxidases (NOXs). NOX enzymes transfer an electron across the cell membrane from NADPH in the cytosol to oxygen (O_2_) in the luminal or extracellular space. This movement of an electron generates O_2_^•−^, which can be rapidly converted into H_2_O_2_ by superoxide dismutases (SODs).

The mammalian NOX family comprises seven members (NOX1–5 and DUOX1–2), which have marked differences in tissue distribution and play a variety of physiological roles (10, 11). Members of this family are also expressed in mammalian ovaries. *Nox4* and *Nox5*, for example, are expressed in human granulosa cells (12). NOX4 and its accessory proteins in human granulosa cells show age-dependent reductions in protein expression, which correlates with low fertility (13). Importantly, pharmacological inhibition of NOX enzymes blocks follicle-stimulating hormone-induced oocyte maturation in mouse cumulus–oocyte complex in vitro (14). Despite these observations, a role for NOX in mammalian ovulation has not been demonstrated.

The NOX family of enzymes is evolutionarily conserved across species (15). The *Drosophila* genome contains one *Nox* gene encoding NOX and one *Duox* gene encoding DUOX. DUOX has an additional peroxidase domain and has been well studied in gut–microbe interaction, wing formation, and wound healing (16–18). Much less is known about *Nox*. Earlier work reported that *Nox* regulates ovarian muscle contraction, which somehow influences ovulation (19). However, the mechanism of NOX regulation of ovulation and the cellular localization of NOX in *Drosophila* remain unclear.

Recent work challenges the concept that ovulation is controlled by ovarian muscle contraction in *Drosophila*. Instead, *Drosophila* ovulation involves active proteolytic degradation of the follicle wall and follicle rupture and shares much in common with mammalian ovulation. Like in mammals, each oocyte in *Drosophila* is encapsulated in a layer of somatic follicle cells to form an egg chamber, which develops through 14 distinct stages to become a mature follicle (stage-14 egg chamber) in ovarioles (20). In mature follicles, the zinc finger transcription factor Hindsight (HNT) induces the expression of matrix metalloproteinase 2 (MMP2) in posterior follicle cells and octopamine receptor in mushroom body (OAMB) in all follicle cells (21). During ovulation, octopamine (OA) is released from neuron terminals in the ovary and binds to its receptor OAMB in stage-14 follicle cells. OAMB receptor activation causes an increase in intracellular calcium that activates MMP2 enzymatic activity, which breaks down posterior follicle cells and induces follicle rupture (22, 23). Strikingly, the entire process of follicle rupture can be recapitulated ex vivo by culturing isolated mature follicles with OA in the absence of ovarian muscles and oviducts (23). This work casts doubt on the proposed involvement of ovarian muscles in follicle rupture/ovulation.

In this study, we investigated the role of *Nox* in *Drosophila* ovulation. To our surprise, we found that ovarian muscle *Nox* does not play a major role in ovulation but rather that *Nox* is enriched in mature follicle cells and is essential for follicle rupture/ovulation. OA/OAMB-Ca^2+^ signaling activates NOX enzymatic activity to produce extracellular O_2_^•−^, which is converted into H_2_O_2_ by an extracellular SOD3. Our results suggest that NOX-produced ROS in mature follicles play a conserved role in regulating follicle rupture/ovulation across species.

## Results

### NOX Functions in Mature Follicle Cells for *Drosophila* Ovulation.

Previous work indicated that NOX functions in ovarian muscles to control muscle contraction and ovulation (19). However, a careful examination of the Gal4 drivers used previously (*SI Appendix*, Fig. S1) and our observation of almost-normal egg laying by females with *Nox* knockdown in muscles (*SI Appendix*, Table S1) indicated that ovarian muscle NOX does not likely play a major role in ovulation. Microarray and RNA-sequencing analysis (24, 25) showed that *Nox* is enriched in stage-13/14 egg chambers but not in activated oocytes (*SI Appendix*, Fig. S2*A*). RT-PCR analysis of isolated follicle cells and oocytes from mature follicles (*SI Appendix*, Fig. S2*B*) further supports that *Nox* is enriched in follicle cells.

**Fig. 1.**
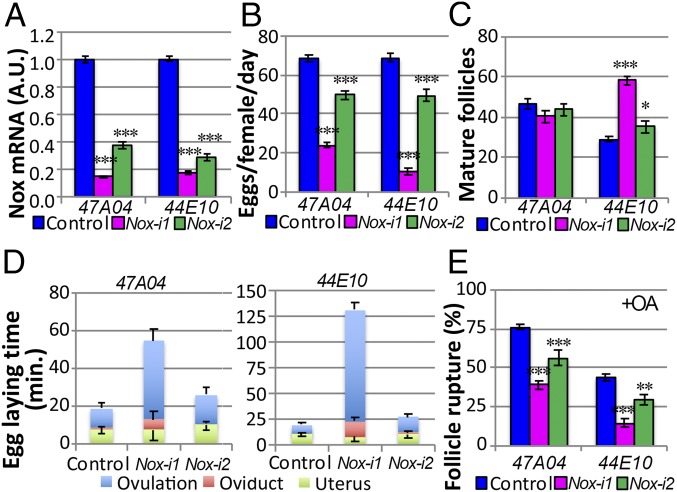
NOX functions in mature follicle cells for ovulation. (*A*) qRT-PCR quantification of *Nox* mRNA in mature follicles from females of control and *Nox-i* driven by *47A04-Gal4* or *44E10-Gal4.* (*B*) Quantification of egg laying from control and *Nox-i* females. Also see *SI Appendix*, Table S2 for the number of females analyzed. (*C*) Quantification of mature follicles in each female’s ovaries after egg laying. The numbers of females used in each genotype are 44, 24, 43, 73, 48, and 42. (*D*) The egg-laying time in control or *Nox-i* females driven by *47A04-Gal4* (*Left*) or *44E10-Gal4* (*Right*). Also see *SI Appendix*, Table S2. (*E*) Quantification of follicle rupture after 3-h culture with 20 μM OA. The numbers of mature follicles used in each genotype are 816, 601, 275, 569, 330, and 387. *P < 0.05, **P < 0.01, ***P < 0.001. *Nox-i*, *Nox-RNAi.*

To probe the function of follicular NOX in late oogenesis and ovulation, we knocked down *Nox* in mature follicle cells. We used two independent RNA interference (RNAi) lines driven by two well-characterized Gal4 drivers, *47A04-Gal4* and *44E10-Gal4* (21–23, 26). *44E10-Gal4* is specifically expressed in follicle cells of all stage-14 egg chambers, whereas *47A04-Gal4* is only expressed in follicle cells of late-stage-14 egg chambers (21). Both *Nox-RNAi* lines significantly reduced *Nox* mRNA levels in mature follicles, with *Nox-RNAi1* showing a more potent reduction (Fig. 1*A*). Females expressing *Nox-RNAi* were subjected to an egg-laying assay and showed a significant reduction in their ability to lay eggs, indicating that *Nox* in mature follicle cells is required for efficient egg laying (Fig. 1*B*). This egg-laying defect in *Nox*-knockdown females is not likely to be due to an oogenesis problem, as ovaries from these females contained normal or even higher numbers of mature follicles (Fig. 1*C*).

Next, we examined whether *Nox*-knockdown females are defective in ovulation and/or oviposition (the process of laying down eggs). Females with *Nox* knockdown (particularly with *Nox-RNAi1*) took a much longer time to ovulate than control females, indicating an ovulation defect (Fig. 1*D* and *SI Appendix*, Table S2). Together, these data suggest that *Nox* in mature follicle cells is required for normal ovulation.

To determine whether *Nox* regulates follicle rupture, a process induced by follicular OA/OAMB signaling during ovulation (23), we cultured *Nox*-knockdown follicles ex vivo by OA stimulation. Consistent with previous results (21), control follicles isolated based on *47A04* and *44E10* expression showed 76% and 39% rupture, respectively, after a 3-h culture with OA (Fig. 1*E*). The difference in rupture rate is due to the fact that *47A04* is expressed only in fully matured follicles (21). By contrast, *Nox*-knockdown follicles showed a significant reduction in OA-induced follicle rupture (Fig. 1*E* and *SI Appendix*, Fig. S2 *C*–*H*), indicating that *Nox* is required for normal follicle rupture. Consistent with this conclusion, pretreatment of mature follicles with diphenyleneiodonium (DPI) or VAS2870, potent NOX enzymatic inhibitors (27), was sufficient to inhibit OA-induced follicle rupture in a dose-dependent manner (*SI Appendix*, Fig. S2 *I* and *J*). Furthermore, the addition of butylated hydroxyanisole (BHA), a broad-spectrum ROS scavenger, in the culture medium also inhibited OA-induced follicle rupture (*SI Appendix*, Fig. S2*K*). Together, these data suggest that NOX functions in mature follicle cells to promote OA-induced follicle rupture and ovulation.

### NOX Does Not Interfere with the OA/OAMB-Ca^2+^–MMP2 Pathway.

**Fig. 2.**
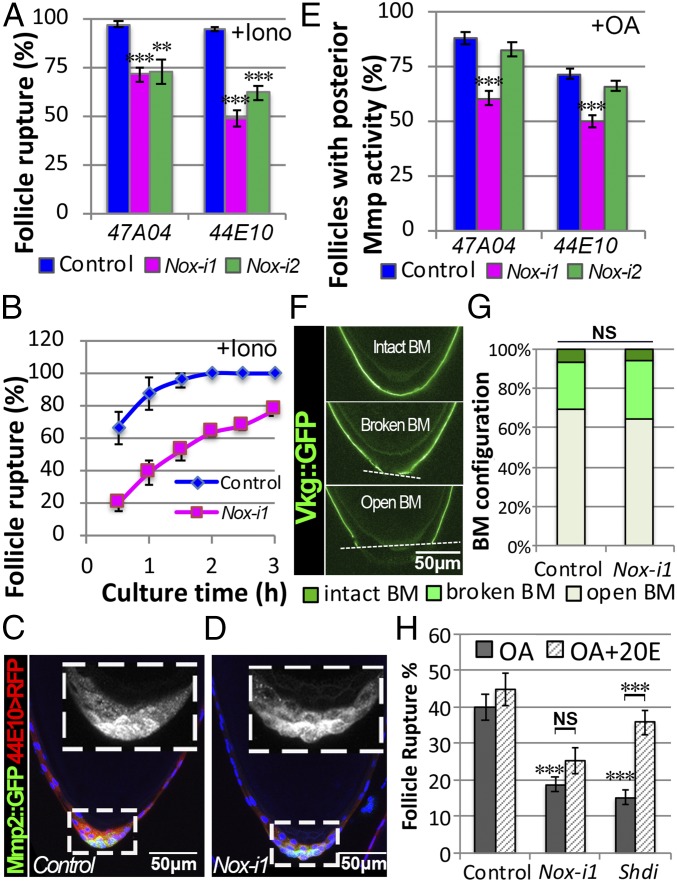
NOX does not interfere with the OA/OAMB-Ca^2+^–MMP2 pathway. (*A*) Quantification of follicle rupture after 3-h culture with 5 μM ionomycin. The numbers of follicles used in each genotype are 199, 134, 111, 446, 357, and 268. (*B*) Cumulative follicle rupture in 3 h in response to ionomycin stimulation. Mature follicles were isolated according to *47A04-Gal4* and three groups of each genotype (∼90 follicles) were used. (*C* and *D*) Representative images show MMP2::GFP expression (green in *C* and *D* and white in *Insets*) in mature follicles of control (*C*) and *Nox-i1* (*D*) driven by *44E10-Gal4*. The mature follicle cells are marked by *44E10-Gal4* driving *UAS-RFP* (*44E10>RFP*; red in *C* and *D*). Only the posterior portions of the follicles are shown. DAPI (blue in *C* and *D*) is used to mark nuclei. (*E*) Quantification of posterior MMP activity in control and *Nox-i* mature follicles with *47A04-Gal4* or *44E10-Gal4* after 3-h culture with OA using in situ zymography. The numbers of mature follicles used in each genotype are 556, 539, 322, 478, 466, and 259. (*F*) Representative images show three categories of BM configurations (according to Vkg::GFP expression in green) in isolated mature follicles. (*G*) Quantification of BM configuration of isolated mature follicles from control or *Nox-i1* females with *44E10-Gal4*. (*H*) Quantification of follicle rupture after treatment with or without 20 nM 20E for 30 min followed by a 6-h OA culture. The numbers of mature follicles used in each genotype are 327, 355, 324, 367, 169, and 226. **P < 0.01, ***P < 0.001. BM, basement membrane; Iono, ionomycin; *Nox-i*, *Nox-RNAi*.

OA/OAMB signaling in mature follicle cells leads to an intracellular Ca^2+^ rise and MMP2 activation (23). To determine whether NOX functions upstream of the Ca^2+^ rise in the OA/OAMB-Ca^2+^–MMP2 pathway, we used ionomycin, a potent Ca^2+^ ionophore, to stimulate follicle rupture directly. More than 90% of control follicles ruptured after a 3-h ionomycin stimulation, in contrast to 70% (in the case of *47A04*) and 40–60% (in the case of *44E10*) of *Nox*-knockdown follicles (Fig. 2*A* and *SI Appendix*, Fig. S3 *A*–*F*). This defect was more obvious when examined before the end of the 3-h culture (Fig. 2*B*). These data suggest that *Nox* regulates molecules downstream of Ca^2+^ in the OA/OAMB-Ca^2+^–MMP2 pathway, or alternatively that *Nox* regulates a different pathway for follicle rupture that is independent from MMP2.

To differentiate between these two hypotheses, we measured MMP2 expression and activation in *Nox*-knockdown follicles. MMP2 protein is properly expressed in posterior follicle cells of *Nox*-knockdown egg chambers (Fig. 2 *C* and *D*). In situ zymography showed that *Nox*-knockdown follicles had slightly reduced MMP activation following OA stimulation (Fig. 2*E* and *SI Appendix*, Fig. S3 *G*–*L*); however, there were no differences in collagen IV [a target of MMP2 (21), encoded by *Viking* (*Vkg*)] between control and *Nox*-knockdown follicles (Fig. 2 *F* and *G*). These data suggest that MMP2 is unlikely to be a major downstream effector of NOX in the follicle rupture process. Consistent with this, *Mmp2* mRNA and genes regulating *Mmp2* expression and activation, including *Oamb* and *Hnt* (21), were not down-regulated in *Nox*-knockdown follicles (*SI Appendix*, Fig. S3*M*).

ROS regulate steroid progesterone production during mammalian ovulation (7). In addition, parallel ecdysteroid signaling is required for *Drosophila* ovulation (26). To determine whether NOX interferes with ecdysteroid production in mature follicle cells, we attempted to rescue the rupture defect of *Nox*-knockdown follicles with 20-hydroxyecdysone (20E). As previously reported, the addition of 20E partially rescues the defect of *shd*-knockdown follicles (26), which lack the ability to convert E to 20E. By contrast, the addition of 20E had no effect on the ability of *Nox*-knockdown follicles to respond to OA-induced rupture (Fig. 2*H*). It is thus unlikely that NOX affects 20E production. In addition, receptors for ecdysteroid signaling were not affected in *Nox*-knockdown follicles (*SI Appendix*, Fig. S3*N*). Given that ecdysteroid signaling strongly interferes with OA-induced MMP2 activation, we believe that NOX does not interfere with ecdysteroid signaling. Together, these data suggest that NOX regulates an unidentified target/pathway for follicle rupture.

### OA Activates NOX in Mature Follicle Cells to Produce Superoxide.

**Fig. 3.**
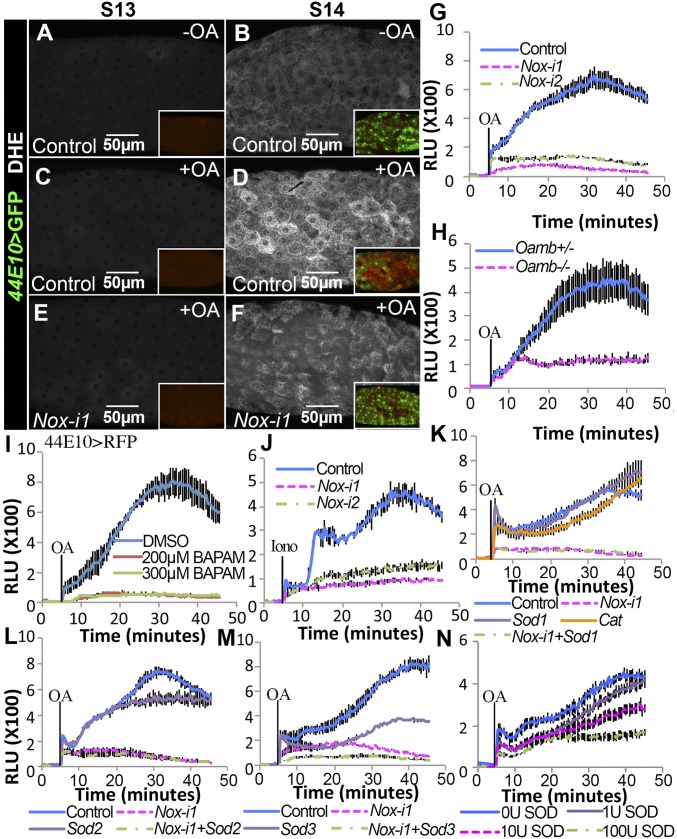
OA activates NOX to produce superoxide extracellularly. (*A*–*F*) Representative images show DHE staining (white in *A*–*F*) in control (*A*–*D*) and *Nox-i1* (*E* and *F*) follicles after 30-min culture without (*A* and *B*) or with (*C*–*F*) OA stimulation. The *Insets* are low-magnification images with *44E10>GFP* expression (green, marking stage-14 follicles) and DHE staining (red). (*G*–*N*) l-012 Luminescence-dependent O_2_^•−^ quantification in mature follicles stimulated with OA (*G*–*I* and *K*–*N*) or ionomycin (*J*) at the 5-min time point. Mature follicles with different genotypes were isolated according to *44E10>RFP* expression. Mature follicles in *I* were pretreated with BAPTA-AM for 30 min before l-012 detection. Mature follicles in *N* were supplemented with SOD extract from bovine erythrocytes in the culture medium. Iono, ionomycin; *Nox-i*, *Nox-RNAi*; RLU, relative luminometer unit.

Although NOX does not interfere with the OA/OAMB-Ca^2+^–MMP2 pathway, OA/OAMB signaling may still regulate the enzymatic activity of NOX, as its N-terminal region contains EF-hand domains for Ca^2+^ binding. To test this hypothesis, we examined O_2_^•−^ production in follicle cells upon OA stimulation. The fluorescent signal of dihydroethidium (DHE), a specific O_2_^•−^ indicator (28, 29), was dramatically increased in stage-14 follicle cells throughout the entire egg chamber after OA stimulation, but not in stage-13 follicle cells (Fig. 3 *A*–*D*). This increase was blocked in *Nox*-knockdown follicle cells (Fig. 3 *E* and *F*). To quantify O_2_^•−^ production in mature follicles, we developed a luminescence assay based on the dye l-012, which has been used to detect O_2_^•−^ in ovaries previously (19). Consistent with DHE staining, OA induced a sharp increase in O_2_^•−^ production in control follicles, which peaked at ∼30–40 min (Fig. 3*G*). In contrast, the increase in O_2_^•−^ production was significantly dampened in *Nox*-knockdown follicles (Fig. 3*G*) or follicles treated with the NOX inhibitor DPI or the ROS scavenger BHA (*SI Appendix*, Fig. S4*A*). In addition, when we used entire ovaries to measure OA-induced O_2_^•−^ production, *Nox* knockdown in mature follicle cells almost completely blocked the OA-induced O_2_^•−^ production (*SI Appendix*, Fig. S4*B*). This finding indicates that OA-induced O_2_^•−^ production is mainly restricted to mature follicle cells and depends on NOX. Thus, these data suggest that OA activates NOX in mature follicle cells to generate O_2_^•−^. Not surprisingly, OA-induced O_2_^•−^ production required OAMB (Fig. 3*H*). In addition, chelating the intracellular Ca^2+^ with 1,2-Bis(2-aminophenoxy)ethane-*N*,*N*,*N′*,*N′*-tetraacetic acid tetrakis(acetoxymethyl ester) (BAPTA-AM) blocked OA-induced O_2_^•−^ production (Fig. 3*I*), and ionomycin was sufficient to induce O_2_^•−^ production in a NOX-dependent manner (Fig. 3*J*). These results suggest that follicular adrenergic signaling induces an intracellular Ca^2+^ rise, which activates NOX enzymatic activity in all mature follicle cells, in addition to MMP2 enzymatic activity in posterior follicle cells, during *Drosophila* ovulation.

### NOX Functions to Produce Superoxide Extracellularly.

It is unknown where NOX is localized subcellularly in mature follicle cells, as a NOX antibody is not available. To probe where NOX is localized to produce O_2_^•−^ for follicle rupture, we overexpressed three distinct *Sods*—cytoplasmic *Sod1* (30), mitochondrial *Sod2* (31), and extracellular *Sod3* (32, 33)—in mature follicle cells to dismutate O_2_^•−^ into H_2_O_2_. Superoxide can hardly diffuse through cell membranes; thus, subcellularly localized SOD is required to dismutate O_2_^•−^. Overexpression of *Sod1* in mature follicle cells did not reduce the amount of O_2_^•−^ generated by OA stimulation (Fig. 3*K*), nor did overexpression of *Sod2* (Fig. 3*L*). In contrast, overexpression of *Sod3* significantly reduced the amount of OA-induced O_2_^•−^ in mature follicles (Fig. 3*M*). We also confirmed that ectopic SOD3 is indeed secreted into the extracellular space (*SI Appendix*, Fig. S5 *A*–*C*). Furthermore, the addition of SOD extract from bovine erythrocytes in the culture medium was sufficient to reduce OA-induced O_2_^•−^ in a dose-dependent manner (Fig. 3*N*). These data not only confirm the specificity of l-012 for O_2_^•−^ detection but also suggest that NOX produces extracellular O_2_^•−^, which can be dismutated by extracellular SOD3 but not cytoplasmic SOD1 or mitochondrial SOD2.

### H_2_O_2_, but Not Superoxide, Is the Key Signaling Molecule for Follicle Rupture.

**Fig. 4.**
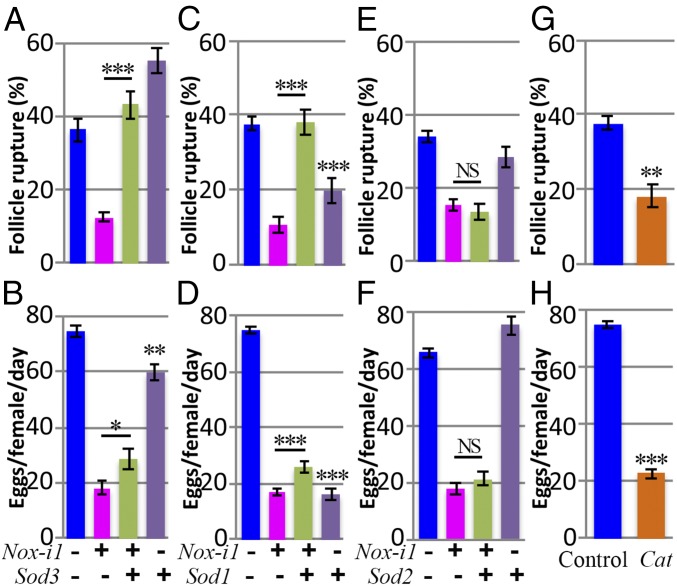
H_2_O_2_ but not superoxide is the key signaling molecule for follicle rupture. (*A* and *B*) Quantification of OA-induced follicle rupture (*A*) and egg laying (*B*) using females with *44E10-Gal4* driving *Nox-i1* and/or *Sod3::3xHA* expression. The numbers of follicles used in *A* are 349, 354, 325, and 283, while the numbers of females used in *B* are 45, 50, 40, and 20. (*C* and *D*) Quantification of OA-induced follicle rupture (*C*) and egg laying (*D*) using females with *44E10-Gal4* driving *Nox-i1* and/or *Sod1* expression. The numbers of follicles in *C* are 445, 355, 357, and 341, while the numbers of females in *D* are 75, 50, 50, and 50. (*E* and *F*) Quantification of OA-induced follicle rupture (*E*) and egg laying (*F*) using females with *44E10-Gal4* driving *Nox-i1* and/or *Sod2* expression. The numbers of follicles used in *E* are 174, 182, 178, and 162, while the number of females used in *F* is 25 for each genotype. (*G* and *H*) Quantification of OA-induced follicle rupture (*G*) and egg laying (*H*) using females with *44E10-Gal4* driving *Cat* expression. The numbers of mature follicles used in each genotype in *G* are 445 and 256. The number of females used in *H* is 50 for each genotype. *P < 0.05, **P < 0.01, ***P < 0.001. *Nox-i*, *Nox-RNAi*.

Despite the fact that NOX regulates follicle rupture by generating O_2_^•−^, which can be quickly converted to H_2_O_2_ by SOD3, it is still unknown whether O_2_^•−^ or its derivative H_2_O_2_ is the signaling molecule responsible for follicle rupture. We reasoned that if O_2_^•−^ is the signaling molecule for follicle rupture, overexpression of *Sod3* in WT or *Nox*-knockdown follicles, which reduces or further reduces the O_2_^•−^ level (Fig. 3*M*), would lead to defective rupture or an enhanced rupture defect, respectively. By contrast, overexpression of *Sod1* or *Sod2*, which did not affect the O_2_^•−^ level, would have a minimal effect. To our surprise, mature follicles with *Sod3* overexpression alone had normal or even better follicle rupture in response to OA stimulation, and *Sod3* overexpression in the *Nox*-knockdown follicles fully rescued the defect of OA-induced follicle rupture (Fig. 4*A*). This result indicates that H_2_O_2_, but not O_2_^•−^, is likely the signaling molecule for follicle rupture. Unfortunately, *Sod3* overexpression only partially rescued the egg-laying defect of *Nox*-knockdown females (Fig. 4*B*). This could be due to an insufficient amount of O_2_^•−^ converted to H_2_O_2_ to execute normal physiology or because O_2_^•−^ plays other roles in the egg-laying process in addition to being converted to H_2_O_2_ for follicle rupture/ovulation.

Consistent with the idea that H_2_O_2_ is the key signaling molecule for follicle rupture, overexpression of *Sod1*, which could produce intracellular H_2_O_2_ to compensate for the loss of NOX/SOD3-generated extracellular H_2_O_2_, exerted a similar rescue effect as *Sod3* (Fig. 4 *C* and *D*). In contrast, overexpression of *Sod2* in mitochondria did not show any rescue effect (Fig. 4 *E* and *F*), indicating that subcellular production of H_2_O_2_ is essential for follicle rupture. Consistent with this, overexpression of *Catalase* (*Cat*), an enzyme converting H_2_O_2_ to H_2_O and O_2_ (34), in mature follicle cells led to a strong reduction in OA-induced follicle rupture and egg-laying number (Fig. 4 *G* and *H*), but did not affect O_2_^•−^ production (Fig. 3*K*). Notably, *Sod1* overexpression alone caused a severe defect in OA-induced follicle rupture and egg laying (Fig. 4 *C* and *D*), indicating that too much intracellular H_2_O_2_ may be toxic for follicle rupture. Not surprisingly, the addition of H_2_O_2_ in the culture medium did not rescue the rupture defect of *Nox*-knockdown follicles (*SI Appendix*, Fig. S5*D*). Taken together, we favor the idea that a spatiotemporal burst of H_2_O_2_ production in the extracellular environment of mature follicle cells is critical for OA-induced follicle rupture.

### SOD3 Is Required to Convert Superoxide to H_2_O_2_ for Follicle Rupture.

**Fig. 5.**
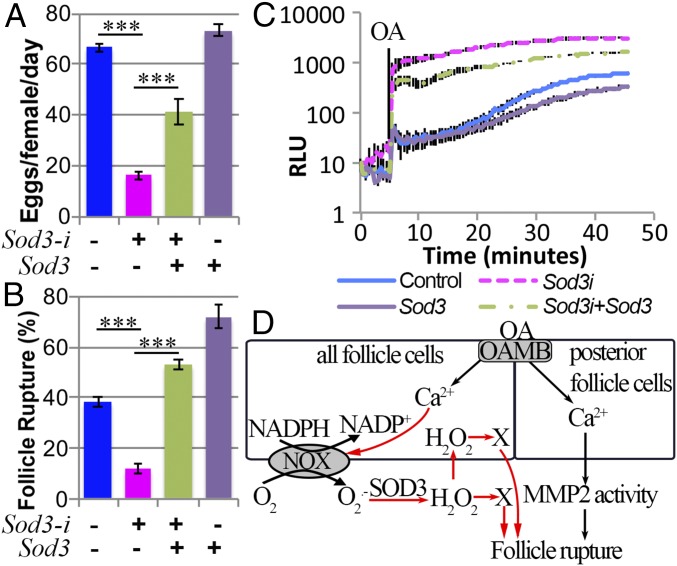
SOD3 in mature follicle cells is required for ovulation. (*A* and *B*) Quantification of egg laying (*A*) and OA-induced follicle rupture (*B*) using females with *44E10-Gal4* driving *Sod3-i* and/or *Sod3::3xHA* expression. Forty to 50 females were used in *A*, and the numbers of follicles used in *B* are 219, 240, 227, and 237. (*C*) l-012 Luminescence-dependent O_2_^•−^ quantification in mature follicles with *44E10-Gal4* driving *Sod3-i* and/or *Sod3::3xHA* expression. Note the *y* axis is different from those in Fig. 3. Mature follicles were stimulated with 20 μM OA at the 5-min point. (*D*) A schematic diagram shows the signaling pathways downstream of the OA/OAMB in mature follicle cells to regulate follicle rupture. ***P < 0.001. RLU, relative luminometer unit.

The above studies indicate that SOD3 likely functions outside the mature follicle cells to convert NOX-produced O_2_^•−^ to H_2_O_2_ to regulate follicle rupture/ovulation. To test this hypothesis, we specifically knocked down *Sod3* in mature follicle cells. Females with *Sod3* knockdown laid <20 eggs/female per day, similar to *Nox*-knockdown females (Fig. 5*A*). In addition, *Sod3*-knockdown mature follicles were defective in OA-induced follicle rupture (Fig. 5*B*). Furthermore, the defective follicle rupture/ovulation in *Sod3*-knockdown females could be significantly rescued by overexpression of *Sod3* (Fig. 5 *A* and *B*). These data suggest that follicular SOD3 is indeed required for follicle rupture/ovulation. As predicted, O_2_^•−^ accumulated fivefold in *Sod3*-knockdown follicles in comparison with control follicles and this accumulation could be partially reduced by overexpression of *Sod3* (Fig. 5*C*). These results further demonstrate that H_2_O_2_, not O_2_^•−^, is responsible for regulating follicle rupture. However, it is unclear whether H_2_O_2_ acts extracellularly or diffuses through the cell membrane to reach its targets for follicle rupture. In conclusion, we identified an OA/OAMB-Ca^2+^–NOX-SOD3 pathway that regulates H_2_O_2_ production and follicle rupture in all mature follicle cells in addition to the previously identified OA/OAMB-Ca^2+^–MMP2 pathway in posterior follicle cells (Fig. 5*D*).

## Discussion

Ovarian ROS are indispensable for ovulation in mice (7). However, the site of production of ROS is unknown and it is unclear whether ROS play a conserved role in ovulation across species. In this study, we provide genetic evidence that follicular ROS are required for ovulation in *Drosophila*. We demonstrate that NOX, whose activity is regulated by follicular adrenergic signaling, regulates follicle rupture and ovulation by producing O_2_^•−^ in the extracellular space of mature follicle cells (Fig. 5*D*). In addition, our data suggest that an extracellular SOD3 converts this O_2_^•−^ into H_2_O_2_, which is the key signaling molecule responsible for regulating follicle rupture (Fig. 5*D*). H_2_O_2_ can partially mimic LH in regulating cumulus expansion and gene expression in mammalian follicles (7). It is thus plausible that H_2_O_2_ plays a conserved role in regulating follicle rupture/ovulation from insects to mammals.

Members of the NOX family are also expressed in mouse and human granulosa cells and are functional in producing ROS (12–14). Norepinephrine, the mammalian counterpart of OA, is highly enriched in human follicular fluid and causes ROS generation in human granulosa cells (35). It will be interesting to determine whether norepinephrine plays a similar role as OA in generating ROS through regulating NOX activity during follicle rupture/ovulation in mammals.

Why would *Drosophila* mature follicles use NOX to generate ROS during follicle rupture? ROS can be generated through the mitochondrial respiratory chain and membrane-bound NOX family enzymes, as well as by a host of intracellular enzymes, such as xanthine oxidase, cyclooxygenases, cytochrome p450 enzymes, and lipoxygenases that produce ROS as part of their normal enzymatic function (36). As high-level cytoplasmic ROS are detrimental to cell function and viability, limiting O_2_^•−^/H_2_O_2_ production in the extracellular environment may be essential for cell viability and function. This is consistent with our finding that overexpression of *Sod1*, which presumably produces extra-cytoplasmic H_2_O_2_, led to a disruption in follicle rupture and egg laying (Fig. 4 *C* and *D*). Interestingly, *Nox*-knockdown follicles overexpressing *Sod1* had normal follicle rupture (Fig. 4*C*), likely due to compensation of NOX-generated H_2_O_2_ by intracellularly produced H_2_O_2_, whereas bathing *Nox*-knockdown follicles in H_2_O_2_ did not rescue the defect in OA-induced follicle rupture (*SI Appendix*, Fig. S5*D*). These findings suggest that local ROS production is essential for cellular physiology, while global ROS may be detrimental.

Interestingly, *Sod3* knockdown alone was sufficient to cause follicle rupture defects in *Drosophila* (Fig. 5 *A* and *B*), yet mice lacking SOD3 are healthy and fertile (37). It is possible that SOD1 can compensate for the loss of SOD3 in mouse follicles, as mice lacking SOD1 or both SOD1 and SOD3 are subfertile or infertile, respectively (38–40).

This study solved a conundrum in *Drosophila* ovulation. Previous work demonstrated that follicle rupture requires OA/OAMB induction of MMP2 activity in posterior follicle cells. However, OA/OAMB induces a rise in intracellular Ca^2+^ in all mature follicle cells (22, 23). What is the role of OA/OAMB-Ca^2+^ in nonposterior follicle cells? Our work demonstrated that OA/OAMB-Ca^2+^ signaling activates NOX in all follicle cells to produce O_2_^•−^ and H_2_O_2_, which are important for follicle rupture (Fig. 3 *A*–*F*). NOX-generated ROS had a minimal effect on MMP2 activity, implying that these ROS regulate an independent pathway that is required for follicle rupture (Fig. 5*D*). Further studies should test whether region-specific *Nox* knockdown, such as only in nonposterior follicle cells, causes a follicle rupture defect.

The targets of H_2_O_2_ in regulating follicle rupture are still unknown. Biological redox reactions catalyzed by H_2_O_2_ typically affect protein function by promoting the oxidation of cysteine residues (41). The best-characterized examples of H_2_O_2_-mediated signal transduction include several protein tyrosine phosphatases in growth factor signaling pathways, such as platelet-derived growth factor, epidermal growth factor (EGF), insulin, and B cell receptor signaling (36, 41, 42). Oxidation of the cysteine residue in the active-site motif of these phosphatases reversibly inactivates phosphatase activity and promotes growth factor signaling. The timing of H_2_O_2_ production and follicle rupture makes it unlikely that H_2_O_2_ promotes follicle rupture in *Drosophila* follicle cells by regulating growth factor signaling. The peak production of O_2_^•−^ (and presumably of H_2_O_2_) is ∼30–40 min after OA stimulation (Fig. 3), which coincides with the beginning of follicle rupture (23). There is not enough time to allow growth factor signaling-mediated transcription and translation to occur before rupture happens. Alternatively, H_2_O_2_ is also involved in the activation of the ADAM (a disintegrin and metalloprotease) family of metalloproteases, possibly through direct oxidation of a cysteine residue that prevents the inhibition of catalytic domain by the prodomain of the enzyme (14, 43, 44). We favor the idea that NOX-generated H_2_O_2_ activates ADAM or other proteinases to regulate follicle rupture in addition to MMP2 activation. Microarray and RNA-sequencing analysis identified multiple proteinases that are up-regulated in *Drosophila* follicle cells during ovulation (24, 25), and at least six different proteinases have been suggested to be involved in mammalian ovulation (45). Recent bioinformatics and large-scale proteomic analyses have predicted >500 proteins containing redox-active cysteine residues (46, 47), some of which could serve as the downstream effectors of H_2_O_2_ for follicle rupture.

## Materials and Methods

Details are described in *SI Appendix*, *SI Materials and Methods*. This includes information on *Drosophila* genetics, egg laying and ovulation time, ex vivo follicle rupture, in situ zymography, qRT-PCR, ROS detection, immunostaining, and microscopy.

## Supplementary Material

Supplementary File
