# Supplementary material for: NADPH oxidase-generated reactive oxygen species in mature follicles are essential for Drosophila ovulation
Source: Proc Natl Acad Sci U S A. 2018 Jul 9;115(30):7765–70. doi: 10.1073/pnas.1800115115 (PMC6065002; doi:10.1073/pnas.1800115115)
Supplement: Supplementary File [file pnas.1800115115.sapp.pdf]

PNAS
www.pnas.org

Supplementary Information for

NADPH oxidase-generated reactive oxygen species in mature follicles are essential for *Drosophila* ovulation

Wei Li, Jessica F. Young and Jianjun Sun

Dr. Jianjun Sun

Email: jianjun.sun@uconn.edu

This PDF file includes:

Supplementary text
Figs. S1 to S5
Tables S1 to S3
References for SI reference citations

www.pnas.org/cgi/doi/10.1073/pnas.1800115115

Supplementary Information Text

SI Results

Previous work used three muscle-Gal4 drivers to demonstrate that NOX functions in ovarian muscles to control muscle contraction and ovulation (1). The three muscle-Gal4 drivers used in the report (*c179*, *c855a*, *Nrv1-Gal4*) were either not at all or not explicitly mapped to the ovarian muscles. Both *c179* and *c855a* are not expressed in ovarian muscles (Fig. S1A-D). Instead, *c179* is expressed in seminar receptacle, spermathecae, and neurons innervating oviduct (Fig. S1A-B). *c855a* is highly expressed in stage-14 follicle cells (Fig. S1C-D). *Nrv1-Gal4* is widely expressed in many tissues in the female reproductive system, including uterus, spermathecae, seminal receptacle, oviduct, follicle cells and ovarian muscles (Fig. S1E-H). These expression patterns made us wonder whether NOX indeed functions in ovarian muscles to control ovulation. To address this question, we used two muscle-specific Gal4 drivers, *act57B-Gal4* and *Mef2-Gal4* (2), to knock down *Nox* and measure the egg-laying potential of these females. *act57B-Gal4* is specifically detected in peritoneal sheath, oviduct and uterus muscle (Fig. S1I-J), while *Mef2-Gal4* is expressed in all muscle cells throughout animal (Fig. S1K-L) (2, 3). Females with *Nox* knockdown in *Mef2-Gal4* cells died gradually after fly enclose, likely due to NOX's role in body muscle for normal physiology. In contract, females with *Nox* knockdown in *act57B-Gal4* cells were viable and laid almost normal number of eggs (Table S1). However, females with *Nox* knockdown in *44E10* or *47A04* cells laid much fewer eggs than control (Table S2). In another experiment, we specifically knocked down *Nox* in adult muscle cells by shifting newly eclosed flies to the restrictive temperature (29°C). These females laid normal number of eggs as control females (Table S1). All these data suggest that ovarian muscle NOX unlikely plays a major role in regulating ovulation as previously reported.

SI Materials and Methods

***Drosophila* genetics**

Flies were reared on standard cornmeal and molasses food at 25°C, and all RNAi knockdown experiments were performed at 29°C with *UAS-dcr2*. Two stage-14 follicle-cell specific Gal4 drivers from the Janelia Gal4 collection (4) were used in this study: *R44E10-Gal4* and *R47A04-Gal4*. The following transgenic lines were used to knock down or overexpression genes in experiments: *UAS-Nox*^{RNAi1} (V4914), *UAS-Sod3*^{RNAi} (V37793), and *UAS-shd*^{RNAi} (V17203) from the Vienna Drosophila Resource Center (VDRC); *UAS-Nox*^{RNAi2} (B32902), *UAS-Sod1* (B24750), *UAS-Sod2* (B27645), and *UAS-Cat* (B24621) from Bloomington Drosophila Stock Center (BDSC); as well as *UAS-Sod3::3XHA* from FlyORF (5). *UAS-RFP* or *UAS-eGFP* was recombined to Gal4 drivers and used for labeling or isolating stage-14 egg chambers for *ex vivo* culture. Protein trap lines *Vkg::GFP*^{CC00791} (6) and *Mmp2::GFP* (7) were used for reporting *Vkg* and *Mmp2* expression, respectively. Control flies for all experiments were prepared from crossing Gal4 drivers to Oregon-R males. *44E10-Gal4, UAS-eGFP, Oamb*^{MI12417}*/TM3, Sb* were crossed to *Df(3R)BSC141/TM3, Sb* (B9501) to generate *Oamb*$^{-/-}$ and *Oamb*$^{+/-}$ females as before (8). For the Gal4 expression mapping, *act57B-Gal4* and *Mef2-Gal4* are kindly provided by Dr. Lynn Cooley (2); *c179-Gal4* (B6450) and *c855a-Gal4* (B6990) are from the BDSC; *Nrv1-Gal4* is kindly provided by Dr. Paul Salvaterra (9). Each Gal4 line was crossed to *UASp-GFP::act79B; UAS-mCD8::GFP*. For flip-out/Gal4 clone analysis in Fig S3N, *hsFLP;; act>CD2>Gal4, UAS-eGFP* were used to cross *UAS-Nox*^{RNAi1}*; UAS-dcr2* males. Adult females with correct genotypes were subjected to a 45-minute heat shock in 37°C water bath to induce flip-out clones before dissection.

Egg laying and ovulation time

Analysis of egg laying and ovulation time was performed as described (8, 10). In short, five- to six-day-old virgin females were fed with wet yeast one day before egg laying, and five females and 10 Oregon-R males were kept in a bottle to lay eggs on molasses-agar plates for two days at 29°C. After egg laying, ovaries were dissected, and mature follicles in these ovaries were quantified. The number of eggs laid on the plates was counted and used to calculate the average time for laying an egg (egg-laying time). The egg-laying time was partitioned into the ovulation time, oviduct time, and the uterus time. The partition ratio was determined based on the percentage of females having eggs in the oviduct or uterus at six hour after mating.

Ex vivo follicle rupture, *in situ* zymography, and quantitative RT-PCR

The *ex vivo* follicle rupture assay and *in situ* zymography were performed as described (8, 10). The detailed protocols can be found (11). In brief, virgin females with *47A04-Gal4* or *44E10-Gal4* driving *UAS-RFP* expression were used to isolate intact mature follicles in Grace's insect medium (Caisson), which were separated into groups of ~30 egg chambers each and cultured in culture media (Grace's medium, 10% fetal bovine serum, and 1X penicillin/streptomycin) supplemented with 20 μM OA (Sigma) or 5 μM ionomycin (Cayman Chemical). For DPI (Cayman Chemical), VAS2870 (Sigma) or BHA (Sigma) treatment, isolated mature follicles were pretreated with indicated chemicals for 30 minutes before adding OA. For *in situ* zymography, 25 μg/mL of DQ-gelatin conjugated with fluorescein (Invitrogen) was supplemented into the culture media. All cultures were performed at 29°C, the same condition as flies were maintained. Ruptured follicles are defined as those oocytes with more than 80% area lacking follicle-cell covering. Mature follicles with posterior fluorescein signal were considered positive for MMP activity. One data point represents the percent of follicles rupture or with MMP activity per experimental group (~30 egg chambers). Data were represented as mean percentage ± standard error (SE); and Student's T-test was used for statistical analysis.

For quantitative RT-PCR, total RNAs were extracted using Direct-zol™ RNA MicroPrep Kit (Zymo Research) from 60 stage-14 egg chambers isolated from ~10 flies. cDNA synthesis, real-time PCR amplification were previously described (12, 10). At least three biological replicates were performed, and data presented as mean ± SE; and Student's T-test was used for statistical analysis. RT-PCR was used to detect *Nox* expression in follicle cells and oocytes. Stage-14 egg chambers were isolated and treated with or without 20 μM OA for three hours. Follicle cells and oocytes from ruptured egg chambers, as well as unruptured egg chambers, were collected for total RNA isolation and RT-PCR analysis. All the primers used for *Rps17*, *Nox*, *Hnt*, *Oamb*, and *Mmp2* are listed in Table S3.

ROS detection

Ovaries were dissected and treated with or without 20 μM OA for 30 minutes in culture media before ROS detection. Dihydroethidium (DHE; Invitrogen/Molecular Probes) labeling of fixed tissue was performed as described with minor modifications (13). In brief, the tissues were incubated with 30 μM DHE for three minutes, washed with Grace's medium for three times (five minutes each), fixed for six minutes in 4% formaldehyde in 1xPBS, rinsed once in 1xPBS right after fixation, and mounted immediately in 1xPBS. Images were acquired using the exact same settings for control and experimental groups.

L-012 (Wako Chemicals) was used for quantifying $O_2^{\bullet-}$ production. Thirty mature follicles isolated as for OA culture were placed in each well of a white 96-well plate with 250 μl culture media plus 200 μM L-012. Plates were placed in a Synergy H1 plate reader (BioTek Instruments, Inc.) and shaked for five seconds before L-012 luminescence reading for 45 minutes with 30-second interval. After 5-minute reading, OA at 20 μM final concentration or ionomycin at 5 μM final concentration were added into each well, shaked for five seconds. Mature follicles were pretreated with BAPTA-AM (Cayman Chemical), DPI, or BHA for 30 minutes before $O_2^{\bullet-}$

detection in some experiments. For SOD treatment, different concentrations of SOD extract from bovine erythrocytes (Sigma) were supplemented in the culture media before $O_2^{\bullet-}$ detection. For $O_2^{\bullet-}$ detection in ovaries, three pairs of ovaries were incubated in each well. Three to four wells/genotype/conditions were used in each experiment, and each experiment was repeated at least twice. The data from one representative experiment were presented as mean ± SE.

Immunostaining and microscopy

Immunostaining was performed following a standard procedure, including ovary dissection, fixation in 4% EM-grade paraformaldehyde for 15 minutes, blocking in PBTG (PBS+ 0.2% Triton X-100+ 0.5% BSA+ 2% normal goat serum), and primary and secondary antibody staining diluted in PBTG. For *Vkg::GFP* analysis, stage-14 egg chambers were first isolated from ovaries in cold Grace's medium before fixation. Mouse anti-GFP (1:2000; Invitrogen), anti-EcR-common, anti-EcR.B1, anti-EcR.A (1:15; all from Developmental Study Hybridoma Bank, DSHB), rabbit anti-GFP (1:4000; Invitrogen), anti-RFP (1:1000; MBL international), and anti-Shd (1: 250; a gift from Michael O'Connor, University of Minnesota Twin Cities, Minneapolis) were used as primary antibodies. Alexa-488, -568 goat anti-mouse and goat anti-rabbit (1:1000, Invitrogen) were used as secondary antibodies. For non-permeable HA antibody staining, 0.2% Triton X-100 was not added in all the steps before washing off HA primary antibody (1:1000; BioLegend), which was incubated with the ovaries for 2 hours at room temperature. After washing off HA antibody, ovaries were blocked again with PBTG for 1 hour, incubated with DE-Cadherin antibody (1:100; DSHB) overnight. HA and DE-Cadherin antibodies were then recognized by Alexa-568 goat anti-mouse and Alexa-488 goat anti-rat secondary antibodies. Images were acquired using a Leica TCS SP8 confocal microscope for immunostaining or Leica MZ10F fluorescent stereoscope with a sCOMS camera (PCO.Edge) for *ex vivo* culture assays, and assembled using Photoshop software (Adobe, Inc.) and ImageJ.

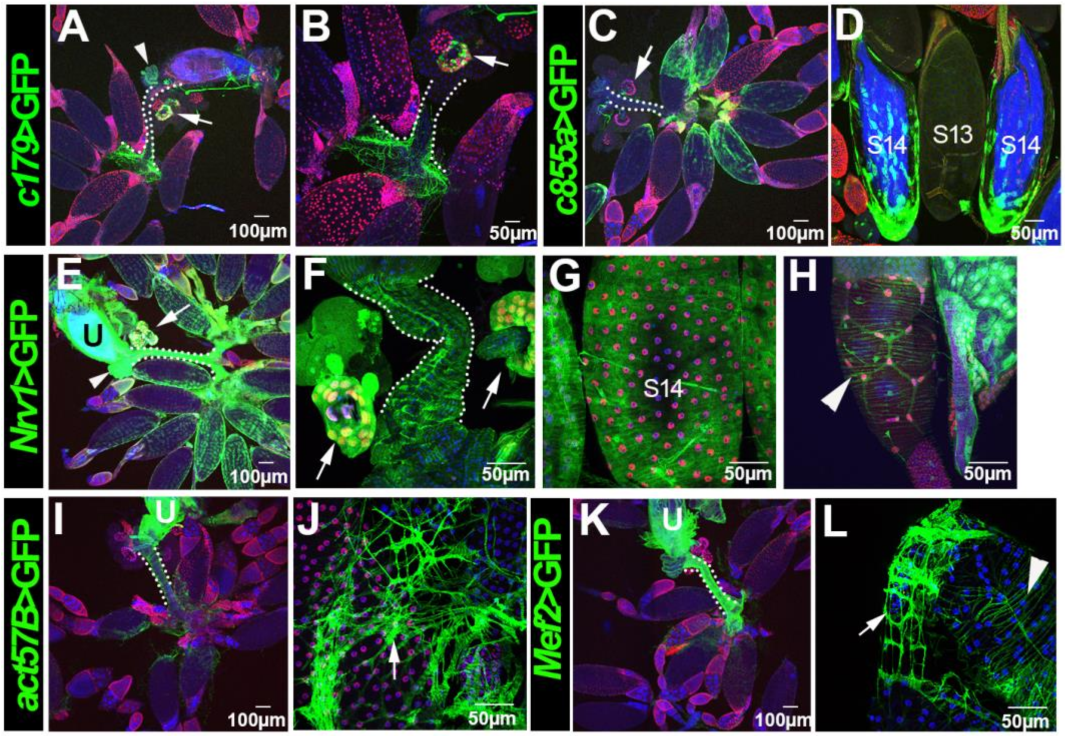

Fig. S1. Gal4 expression in the female reproductive system. Gal4 expression is reflected by *UAS-GFP* expression in green. Hnt expression is shown in red. DAPI (blue) is used to mark nuclei. (A-B) *c179-Gal4* is detected in spermathecae (arrow in A and B), seminal receptacle (arrowhead in A), and neurons innervating the oviduct (outlined in A and B). The B is a higher magnification of part of A. (C-D) *c855a-Gal4* is highly expressed in stage-14 (S14) follicle cells but not in younger follicles. Spermatheca is marked by the arrow and the oviduct is outlined. (E-H) *Nrv1-Gal4* is widely expressed in uterus (marked with U in E), seminal receptacle (arrowhead in E), spermathecae (arrows in E and F), oviduct (outlined in E and F), follicle cells (G) and ovariole sheath (arrowhead in H). (I-J) *act57B-Gal4* is detected in the uterus (marked with U in I), oviduct (outlined in I), and ovarian peritoneal sheath (arrow in J). (K-L) *Mef2-Gal4* is expressed in uterus (marked with U), oviduct (outlined in K), ovariole sheath (arrowhead in L), and peritoneal sheath (arrow in L).

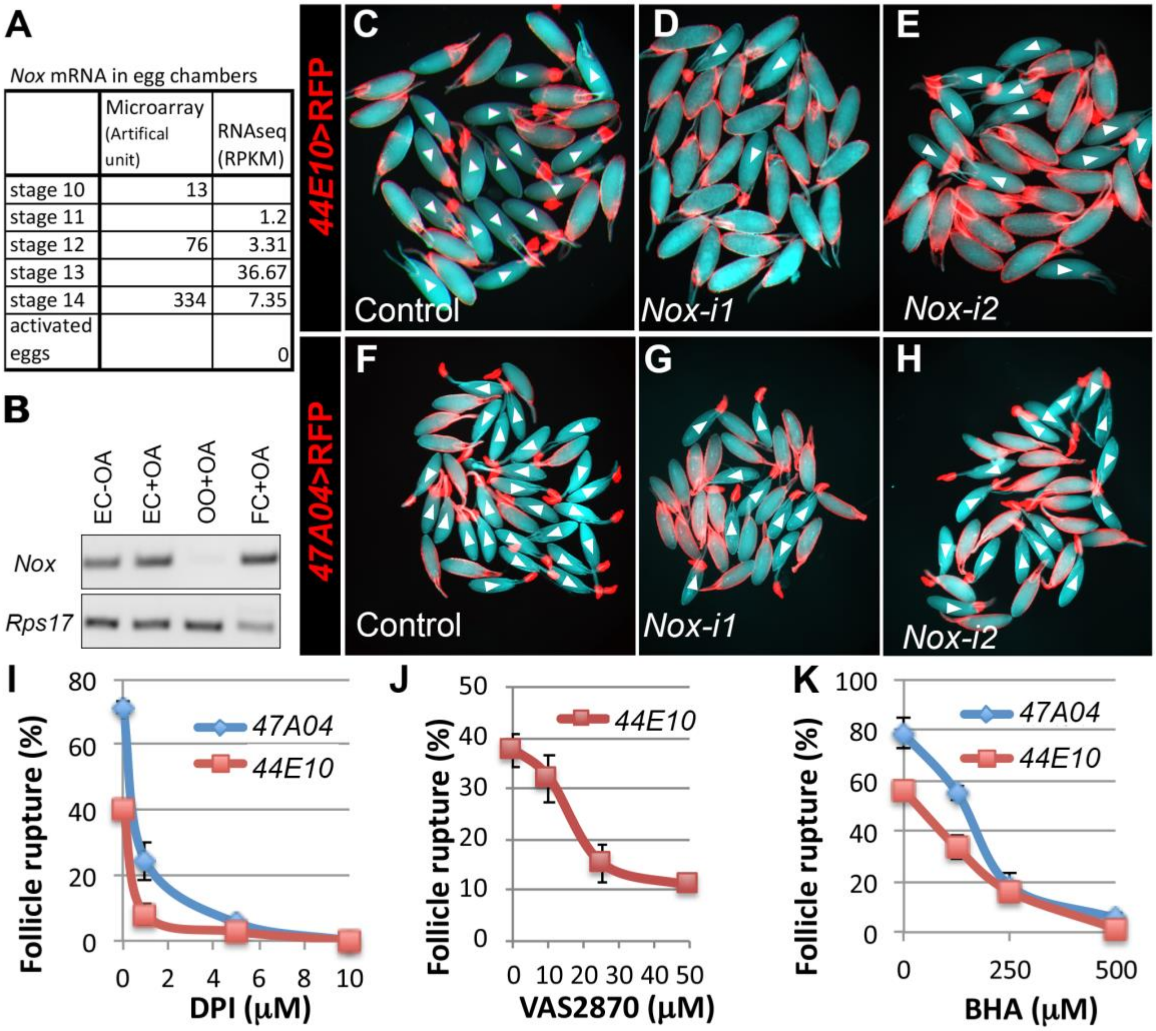

Fig. S2. *Nox* expression in egg chambers and its role in OA-induced follicle rupture. (A) *Nox* mRNA level in egg chambers of different stages. Data were mined from microarray (14) and RNAseq (15) analysis. (B) RT-PCR analysis of *Nox* mRNA level in egg chambers without OA stimulation (EC-OA), unruptured egg chambers with OA stimulation (EC+OA), oocytes (OO+OA) and follicle cells (FC+OA) from ruptured egg chambers with OA stimulation. *Nox* is highly enriched in follicle cells but not in oocytes. The internal control *Rps17* was amplified for 24 cycles, while *Nox* for 35 cycles. (C-H) Representative images show follicles with *44E10-Gal4* (C-E) or *47A04-Gal4* (F-H) driving *Nox-i* expression after three-hour culture with OA. Bright-field images are shown in cyan and RFP is shown in red. Ruptured follicles are marked by arrowheads. (I-K) Quantification of follicle rupture after treatment with different concentrations of DPI (I), VAS2870 (J) or BHA (K) for 30 minutes followed with three-hour OA culture. Mature follicles from control females were used.

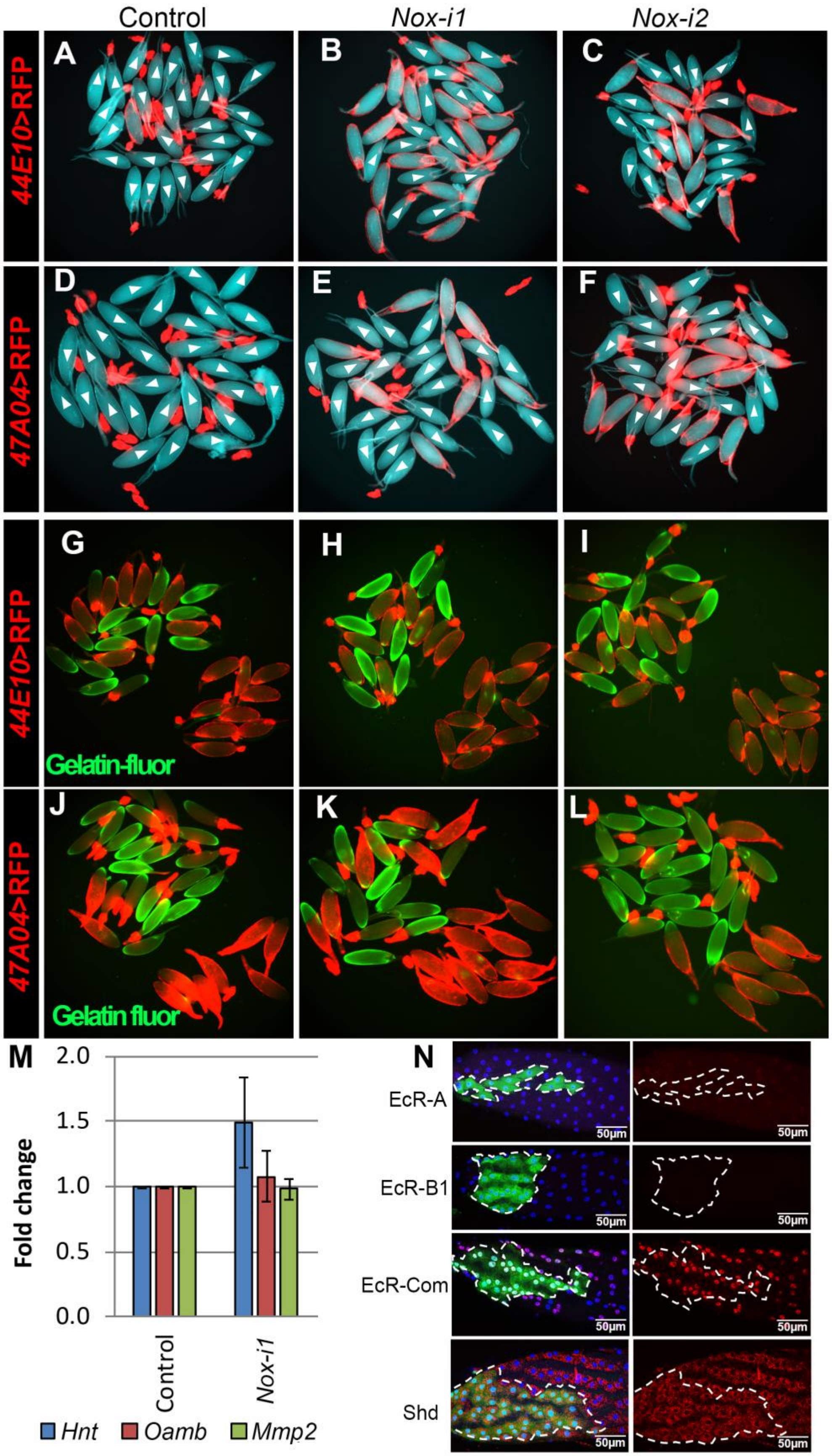
Control
Nox-i1
Nox-i2
44E10>RFP
47A04>RFP
A
B
C
D
E
F
G
H
I
J
K
L
Gelatin-fluor
Gelatin fluor
M
Fold change
2.0
1.5
1.0
0.5
0.0
Control
Nox-i1
Hnt
Oamb
Mmp2
N
EcR-A
EcR-B1
EcR-Com
Shd
50μm

Fig. S3. NOX does not regulate MMP activity. (A-F) Representative images show mature follicles after three-hour culture with ionomycin. Bright-field images are shown in cyan and ruptured follicles are marked by arrowheads. (G-L) Representative images show mature follicles after three-hour culture with OA and fluorescein-conjugated DG-gelatin. Mature follicles with posterior fluorescein signal (green) indicating MMP activity are in the upper panel of each picture. RFP is shown in red. (M) qRT-PCR quantification of *Hnt*, *Oamb* and *Mmp2* mRNA levels in *Nox-i1* mature follicles with *44E10-Gal4*. The data are presented as mean ± SE for three biological replicates. (N) Expressions of EcR-A, EcR-B1, EcR-Com (all isoforms), and Shd (red) are not affected in clonal follicle cells (marked with GFP in green), where *Nox* was knocked down with *act-Gal4* driving *Nox-i1* expression. *Nox*-knockdown clone cells are highlighted by dashed lines. Stage-14 egg chambers are shown. DAPI (blue) was used to mark nuclei, and the single red channel is shown on right panels.

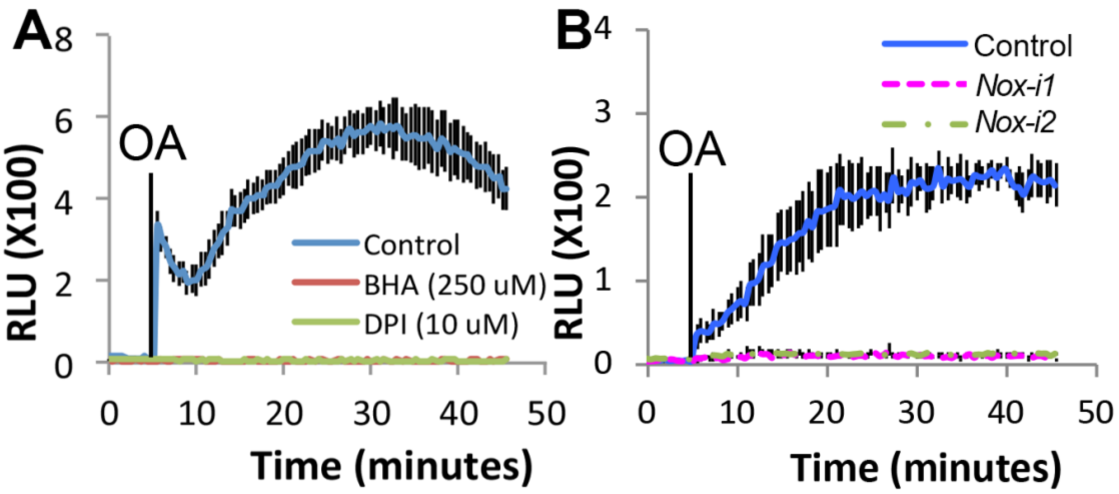

Fig. S4. Superoxide production in mature follicles and ovaries. (A) L-012 luminescence in mature follicles stimulated with OA at the 5-minute point. Mature follicles were isolated according to *47A04>RFP* expression and treated with 250 µM BHA or 10 µM DPI for 30 minutes before L-012 luminescence detection. (B) L-012 luminescence in ovaries stimulated with OA. Ovaries were isolated from control females or females with *44E10-Gal4* driving *Nox-i* expression.

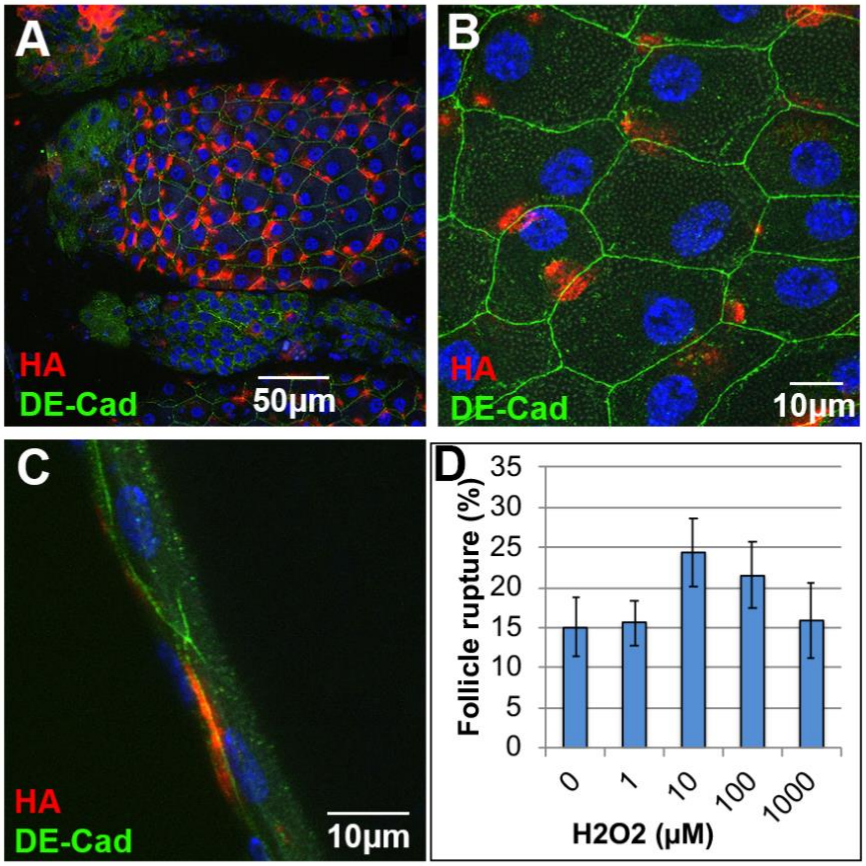

Fig. S5. SOD3 localization and rescuing rupture defect of Nox-knockdown follicles with H_2O_2. (A-C) SOD3::3xHA (red) can be secreted into the extracellular space when overexpressed in mature follicle cells with *44E10-Gal4*. HA staining (red) is used a non-permeable protocol (see SI MM). The adherent junction is marked by DE-Cad staining (green). B is a surface view of the follicle-cell layer, while C is a cross-section view of the follicle-cell layer. DAPI (blue) was used to mark nuclei. (D) Addition of H_2O_2 to the culture medium15 minutes after OA stimulation did not significantly increase the rupture rate of *Nox*-knockdown follicles with *44E10-Gal4* driving *Nox-i1*. The number of follicles used is 202, 274, 251, 225 and 196.

Table S1. The egg-laying effect of *Nox* knockdown with muscle-specific Gal4 lines

Genotype	Egg laying in 2 days#	
	N	Eggs/ female/ day
+/+; UAS-dcr2/+; act57B-Gal4/+	*10*	*63.60±5.54*
+/+; UAS-dcr2/UAS-Nox^{i1}; act57B-Gal4/+	*25*	*51.24±1.97**
+/+; UAS-Nox^{i1}/+; UAS-dcr2/+	*25*	*65.68 ± 2.62*
tubGal80^{ts}/+; UAS-Nox^{i1}/+; Mef2-Gal4/UAS-dcr2	*25*	*58.92±0.87*
+/+;UAS-Nox^{i1}/tubGal80^{ts}; act57B-Gal4/UAS-dcr2	*25*	*68.44± 0.70*

# one day =22 h at 29 °C

* P<0.05, *** P<0.001

Flies with *tubGal80^{ts}* were raised at room temperature and shifted to restrictive temperature (29 C) upon fly enclose. All data are mean ± 95% confidence interval. Student's T-test was used.

Table S2. The effect of *Nox* on egg laying, egg distribution in the reproductive tract, and egg-laying time.

Genotype	Egg laying in 2 days[#]		Egg distribution in 6h			Egg laying time (min)			
	N	Eggs/ female/ day	N	Uterus with egg (%)	Oviduct with egg (%)	Total time	Ovulation time	Oviduct time	Uterus time
UAS-dcr2/+; 47A04-Gal4, UAS-RFP/+	100	68.7 ± 0.7	56	42.9 ± 13.0	1.8 ± 3.5	19.2 ± 0.2	10.6 ± 2.5	0.3 ± 0.7	8.2 ± 2.5
UAS-dcr2/UAS-Nox^{i1}; 47A04-Gal4, UAS-RFP/+	50	24.0 ± 1.0***	51	13.7 ± 9.4	9.8 ± 8.2	54.9 ± 2.2***	42 ± 6.6***	5.4 ± 4.5	7.5 ± 5.2
UAS-dcr2/+; 47A04-Gal4, UAS-RFP/UAS-Nox^{i2}	75	49.8 ± 1.2***	56	39.3 ± 12.8	1.8 ± 3.5	26.5 ± 0.6***	15.6 ± 3.4	0.5 ± 0.9	10.4 ± 3.4
UAS-dcr2/+;44E10-Gal4, UAS-RFP/+	40	68.9 ± 1.6	79	55.7 ± 11.0	2.5 ± 3.5	19.1 ± 0.4	8 ± 2.1	0.5 ± 0.7	10.7 ± 2.1
UAS-dcr2/ UAS-Nox^{i1}; 44E10-Gal4, UAS-RFP/+	50	10.0 ± 1.1***	71	5.6 ± 5.4	11.3 ± 7.4	131.7 ± 14.1***	109.5 ± 16.4***	14.8 ± 9.8	7.4 ± 7.1
UAS-dcr2/+; 44E10-Gal4, UAS-RFP / UAS-Nox^{i2}	50	49.3 ± 1.9***	83	37.3 ± 10.4	6.0 ± 5.1	26.8 ± 1.1***	15.2 ± 2.9	1.6 ± 1.4	10 ± 2.8

# one day =22 h at 29 °C

* P<0.05, ** P<0.01, *** P<0.001

All data are mean ± 95% confidence interval. Student's T-test was used for egg laying, and Z-Score test was used for egg laying time assuming normal distribution.

Table S3. Primers for PCR

Gene Name	Primer Name	Sequence (5'->3')
Rps17	Rps17_RTF	CGAACCAAGACGGTGAAGAAG
	Rps17_RTR	CCTGCAACTTGATGGAGATACC
Nox	Nox_RTF	GAAGCTCTTCCAGCTGTTTGAT
	Nox_RTR	TGAAGGAAACCCTCTTGTTCTC
Hnt	Hnt_RTF	ACATCCGGTGCCACAATTAC
	Hnt_RTR	GTGAACGTCAGGTGGCAGTAG
Oamb	Oamb_RTF	TGACCAACGATCGGGGTTAT
	Oamb_RTR	ATGCGCAATATGAGCTGGGA
Mmp2	Mmp2_RTF	TACTTGTGGCGCATTGGAAC
	Mmp2_RTR	ATCGATGTGGGTCAAAGTGG

References

1. Ritsick DR, Edens WA, Finnerty V, Lambeth JD (2007) Nox regulation of smooth muscle contraction. *Free Radical Biology and Medicine* 43(1):31–38.

2. Hudson AM, Petrella LN, Tanaka AJ, Cooley L (2008) Mononuclear muscle cells in Drosophila ovaries revealed by GFP protein traps. *Developmental Biology* 314(2):329–340.

3. Ranganayakulu G, Schulz RA, Olson EN (1996) Wingless Signaling InducesnautilusExpression in the Ventral Mesoderm of theDrosophilaEmbryo. *Developmental Biology* 176(1):143–148.

4. Pfeiffer BD, et al. (2008) Tools for neuroanatomy and neurogenetics in Drosophila. *PNAS* 105(28):9715–9720.

5. Bischof J, et al. (2013) A versatile platform for creating a comprehensive UAS-ORFeome library in Drosophila. *Development* 140(11):2434–2442.

6. Buszczak M, et al. (2007) The Carnegie Protein Trap Library: A Versatile Tool for Drosophila Developmental Studies. *Genetics* 175(3):1505–1531.

7. Deady LD, Shen W, Mosure SA, Spradling AC, Sun J (2015) Matrix Metalloproteinase 2 Is Required for Ovulation and Corpus Luteum Formation in Drosophila. *PLoS Genetics* 11:e1004989.

8. Deady LD, Sun J (2015) A Follicle Rupture Assay Reveals an Essential Role for Follicular Adrenergic Signaling in Drosophila Ovulation. *PLoS Genet* 11(10):e1005604.

9. Sun B, Xu P, Wang W, Salvaterra PM (2001) In vivo modification of Na+,K+-ATPase activity in Drosophila. *Comparative Biochemistry and Physiology Part B: Biochemistry and Molecular Biology* 130(4):521–536.

10. Knapp E, Sun J (2017) Steroid signaling in mature follicles is important for Drosophila ovulation. *Proceedings of the National Academy of Sciences* 114(4):699–704.

11. Knapp E, Deady L, Sun J (2018) Ex vivo Follicle Rupture and in situ Zymography in Drosophila. *BIO-PROTOCOL* 8(10):e2846.

12. Deady LD, Li W, Sun J (2017) The zinc-finger transcription factor Hindsight regulates ovulation competency of Drosophila follicles. *eLife Sciences* 6:e29887.

13. Owusu-Ansah E, Yavari A, Banerjee U (2008) A protocol for _in vivo_ detection of reactive oxygen species. Available at: http://dx.doi.org/10.1038/nprot.2008.23.

14. Tootle TL, Williams D, Hubb A, Frederick R, Spradling A (2011) Drosophila Eggshell Production: Identification of New Genes and Coordination by Pxt. *PLoS ONE* 6(5):e19943.

15. Eichhorn SW, et al. (2016) mRNA poly(A)-tail changes specified by deadenylation broadly reshape translation in Drosophila oocytes and early embryos. *eLife* 5:e16955.
